# α-Helices in the Type III Secretion Effectors: A Prevalent Feature with Versatile Roles

**DOI:** 10.3390/ijms22115412

**Published:** 2021-05-21

**Authors:** Anastasia D. Gazi, Michael Kokkinidis, Vasiliki E. Fadouloglou

**Affiliations:** 1 Unit of Technology & Service Ultrastructural Bio-Imaging (UTechS UBI), Institut Pasteur, 75015 Paris, France; 2 Institute of Molecular Biology and Biotechnology, Foundation for Research and Technology-Hellas, Nikolaou Plastira 100, Heraklion, 70013 Crete, Greece; kokkinid@imbb.forth.gr; 3 Department of Biology, Voutes University Campus, University of Crete, Heraklion, 70013 Crete, Greece; 4 Department of Molecular Biology & Genetics, Democritus University of Thrace, 68100 Alexandroupolis, Greece

**Keywords:** Type III Secretion System (T3SS), Type III Secretion effector (T3SE), dictionary of secondary structure in proteins (DSSP), 4-α-helix bundle, coiled coil, Novel E3 Ligase (NEL), Transcription Activator-Like Effector (TALE), Leucine-Rich Repeat (LRR)

## Abstract

Type III Secretion Systems (T3SSs) are multicomponent nanomachines located at the cell envelope of Gram-negative bacteria. Their main function is to transport bacterial proteins either extracellularly or directly into the eukaryotic host cell cytoplasm. Type III Secretion effectors (T3SEs), latest to be secreted T3S substrates, are destined to act at the eukaryotic host cell cytoplasm and occasionally at the nucleus, hijacking cellular processes through mimicking eukaryotic proteins. A broad range of functions is attributed to T3SEs, ranging from the manipulation of the host cell’s metabolism for the benefit of the bacterium to bypassing the host’s defense mechanisms. To perform this broad range of manipulations, T3SEs have evolved numerous novel folds that are compatible with some basic requirements: they should be able to easily unfold, pass through the narrow T3SS channel, and refold to an active form when on the other side. In this review, the various folds of T3SEs are presented with the emphasis placed on the functional and structural importance of α-helices and helical domains.

## 1. Introduction

Type III Secretion Systems (T3SSs) are multiprotein nanomachines crossing the three main physical barriers of Gram-negative bacteria: the inner cell membrane, the peptidoglycan layer and the outer bacterial membrane. These systems, originating from the bacterial flagellum, have further evolved and diversified to bypass additional physical barriers: the host cell membrane and, in the case of plant pathogens, the cell wall [1,2,3]. Their ultimate task is to deliver bacterial effector proteins (T3SEs) to the host cell cytoplasm in order to hijack the eukaryotic cell metabolism.

**Figure 1:**
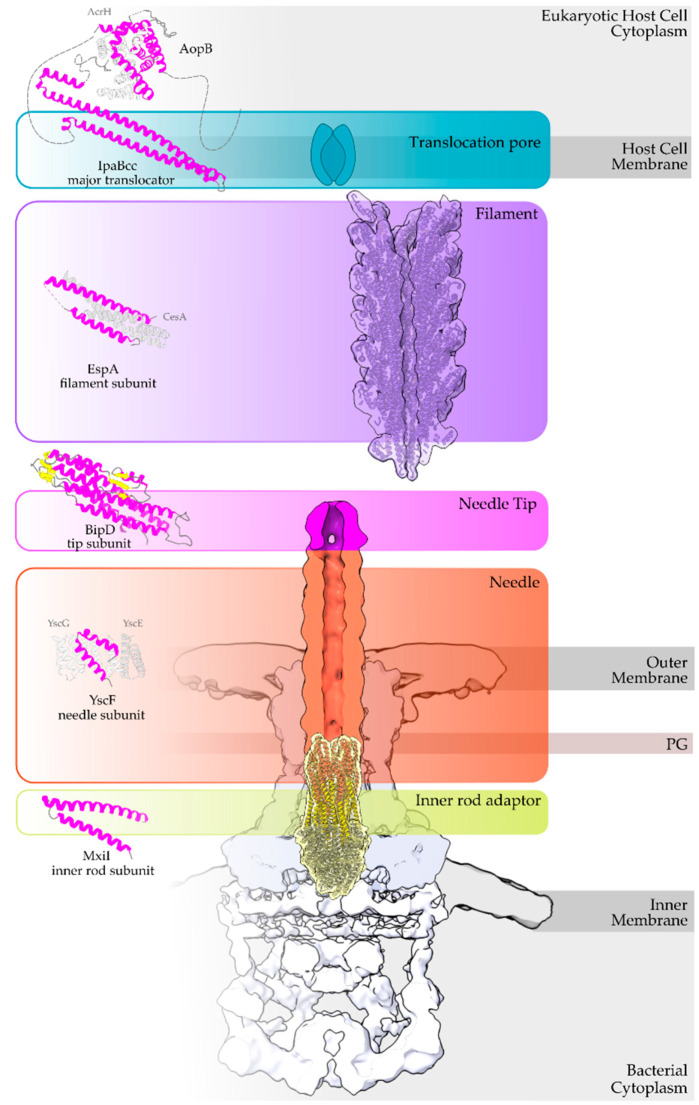
Overview of a T3S nanomachine embedded in the two bacterial membranes. Early and middle secretion substrates are almost all α-helical. The early secretion substrates build the inner rod adaptor (yellow α-helices just above the secretion core, PDB id 6RWY) and the needle (in orange, EMDB id 2803, red α-helices, PDB id 6RWY) or pilus of the nanomachinery that extends to the extracellular space. In animal pathogenic bacteria, the needle is capped with the pentameric needle tip (here in blue, EMDB id 2803), a middle stage substrate. Enteropathogenic (EPEC)/enterohemorrhagic (EHEC) *Escherichia coli* produce thicker extracellular appendages termed filaments (here in purple, PDB id 7KHW). The rest of the parts of the T3S nanomachine, that are not formed from secretion substrates, are depicted in shades of grey to white (PDB id: 6Q15, EMDB ids: 20561, 20611). In the left column, high resolution structures in cartoon representation (α-helices in magenta, β-strands in yellow) corresponding to early secretion substrates that polymerize to build the corresponding T3S parts shown in the right column. Chaperones to maintain early secretion substrates inside the bacterial cytoplasm have also been described (here represented in different shades of grey: light grey for chaperones with tetratrico-peptide repeats (TPR), grey for the rest). Translocators are also considered middle stage secretion substrates as they must be secreted before the effectors. The major subunit of the translocation pore is maintained inside the bacterial cytoplasm in a secretion competent folding state. The chaperone of the translocator, which also possesses TPR, anchors to the N-terminal Chaperone Binding Domain (CBD) of the translocator, while also covering the transmembrane helices of the translocator by extensively interacting with it [13]. IpaBcc denotes the known long coiled coil domain of IpaB. The CBD is proceeding this domain, while the transmembrane helices are following this domain and shown here protected by the chaperone in an analogy to the AcrH/AopB case [14]. PDB ids used for the left column: 3WXX, 5WKQ, 6RWY, 2IZP, 2P58, 1XOU.

The secretion core of these nanomachines and the secretion channel are secured in the bacterial membranes through a series of highly symmetrical rings [4]. The sorting of the secretion substrates is highly regulated, in space and time in response to the environmental signals received, through a large cytoplasmic platform that gates the secretion core [5,6]. The early secretion substrates build the extracellular parts of these machineries, such as the so-called hollow inner rod that extends to the hollow needle or pilus structure (Figure 1). Middle secretion stage substrates then follow. These are, for example, either the helper proteins to break down the plant cell wall and clear the path for the growing pilus, οr the translocator proteins charged with the task to form pores in the host cell membrane. The T3S nanomachinery is then docked to these translocator pores and the secretion of the late secretion substrates, the bacterial Type III Secretion effectors (T3SEs), is finally allowed [7].

T3SEs are an extremely diverse group of proteins. Bacteria usually possess a huge weaponry targeting a vast array of eukaryotic pathways, with some of them acting even inside the host cell nucleus or other cellular compartments [8]. The main goals are to bypass or trick the eukaryotic defense mechanisms and manipulate the host on behalf of the bacterium [9]. T3SEs mimic many eukaryotic proteins in order to trick the pathways they target [10]. T3SEs do not act individually though—they form robust networks. These networks are flexible enough to tolerate effector losses up to 60% without affecting virulence, while the network composition contributes to host adaptability [11]. A large subgroup of T3SEs targeting animal cells have evolved domains able to reorganize the host cell cytoskeleton in order to (a) promote bacterial uptake, (b) hamper the fusion of the bacterial containing vacuole to lysosomes, or (c) use actin tails to freely move the bacterium inside the host cell cytoplasm, to name a few. On the contrary, plant pathogenic bacteria are obliged to a faster compositional turnover of their T3SE networks to accommodate in addition the R protein-mediated secondary defense [12]. As a result, plant pathogenic bacteria end up with a remarkably higher number of T3SE genes in their genome in comparison to their animal pathogenic counterparts.

**Figure 2:**
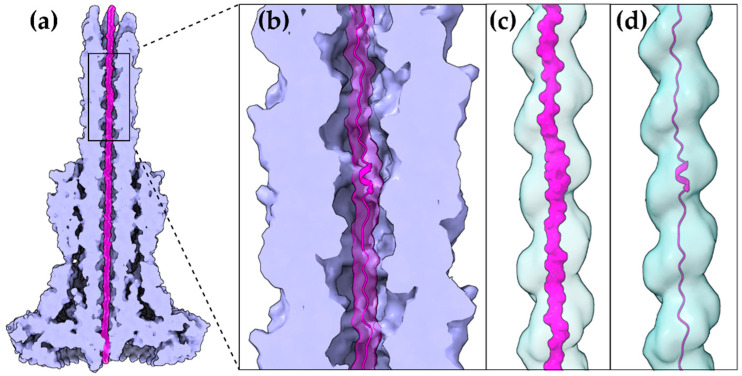
A T3S effector trapped inside the secretion tunnel. The T3SS needle complex atomic structure of *Salmonella enterica* was determined in two functional states. Here with the secretion substrate trapped inside (PDB id 7ahi) [21]. (**a**) A calculated 5 Å map of the atomic model is shown for simplicity. Substrate is displayed in magenta, T3SS needle complex in light purple. (**b**) Zoom on the needle and the secretion tunnel. (**c**,**d**) Smoothed surface of the tunnel displayed in light blue. The tunnel has a right-handed helical grooved surface and is wide enough to allow secretion substrates to form α-helices. In (**c**) the molecular surface representation of the substrate is shown. In (**d**) the cartoon representation is shown.

Despite the different evolutionary pressures applied to animal and plant pathogenic bacteria, some common rules are followed. The bacterium has to produce proteins which successfully mimic eukaryotic ones in order to manipulate the host. These new protein domains should be maintained in a secretion competent folding state when inside the bacterial cytoplasm, delivered successfully to the secretion machinery, easily unfolded to pass through the narrow secretion gate, reach the other end and successfully fold after their release to the eukaryotic cell cytoplasm. To fulfill some of these prerequisites, T3SEs possess N-terminal, usually unstructured, regions with specific characteristic biases and patterns [15]. Moreover, when inside the bacterial cytoplasm, the T3SEs usually form complexes with specialized chaperones through their N-terminal, Chaperone Binding Domain (CBD), until the time comes for the delivery of the substrate to the secretion gating mechanism. The T3SS ATPase is used as the energy source for the release of the chaperone and the subsequent unfolding of the T3SE [16]. Interestingly, not all protein domains can be unfolded successfully by the T3SS ATPase. Chimeras of various domains with N-terminal secretion domains of T3SEs are able to block the secretion and trap these chimeric substrates to the secretion channel [17]. The Proton Motive Force (PMF) is considered to be the driving force of the unfolded polypeptide inside the secretion tunnel [18]. In the case of the bacterial flagellum, PMF provides the energy for the flagellar rotation [19]. However, in the T3SS case, PMF has been proposed to facilitate secretion through needle rotation opposite to the right-handed helical grooved surface of the secretion channel [20,21] (Figure 2), while alternative theories have also been proposed, such as the contribution from the refolding of the effectors, upon exiting the channel, which could pull forward the following polypeptides [18].

**Figure 3:**
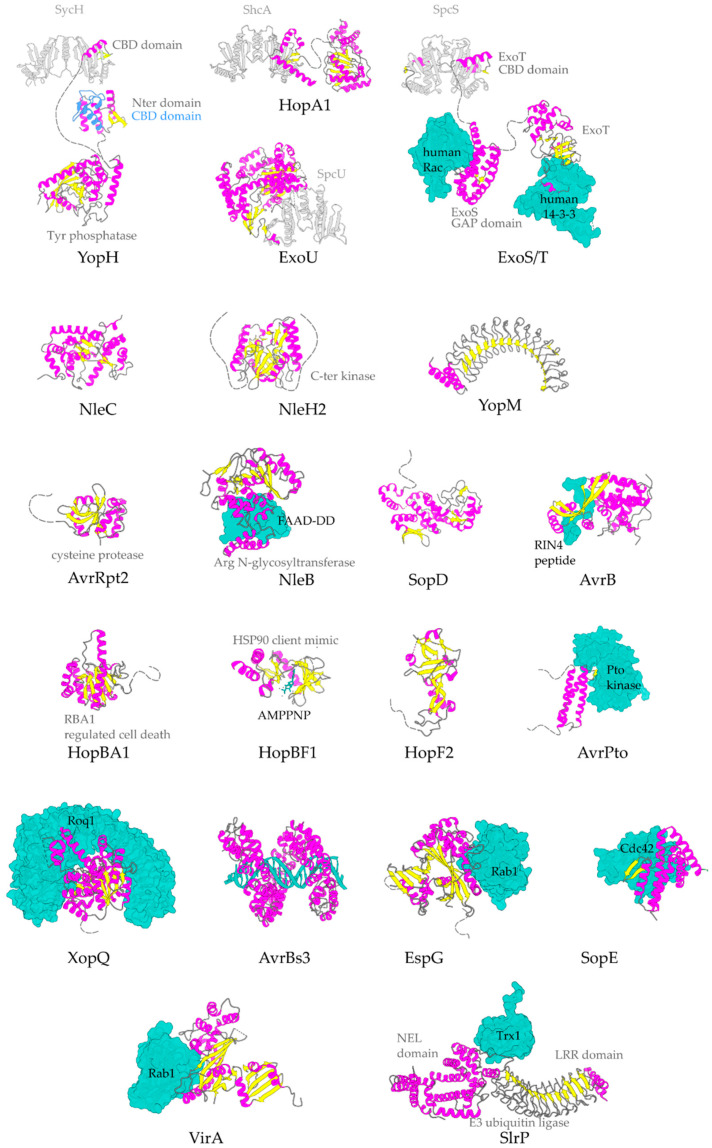
Representative structures for T3SEs. T3SEs are shown in cartoon representation with α-helices in magenta, β-strands in yellow, rest of secondary structure in grey. N-terminal and C-terminal unstructured parts are depicted here in grey broken lines. Dimeric T3SS class I chaperones (or chaperones of effectors) are displayed in grey cartoon representation. Host protein targets that have been co-crystallized with the T3SEs are shown in surface representation in green color. In blue, the alternative conformation of YopH Chaperone Binding Domain (CBD), in the absence of the cognate chaperone. PDB ids used: 1JL5, 4PUF, 4O96, 4O2I, 2QKW, 2NUD, 4FMB, 1GZS, 5CPC, 1S21, 3TU3, 6ACI, 1HE1, 1XXP, 6GNN, 7JLU, 2YPF, 6HQZ, 4RSW, 5T09, 6PWD.

**Figure 4:**
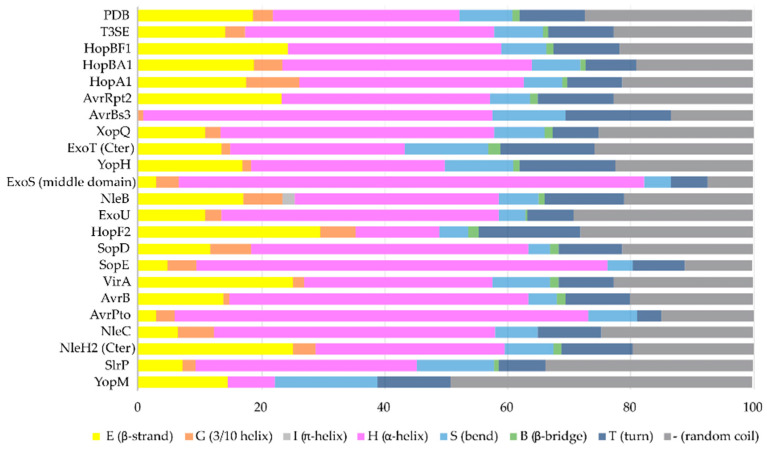
The secondary structure composition of T3SE domains in comparison to PDB. The average occurrence of α-helices in T3SEs is approximately 10% higher compared to the PDB proteome [25]. However, the information from the T3SE-determined crystal structures usually comes from the structured domains of the T3SEs, while their frequently unstructured/flexible N-terminal and C-terminal parts are usually missing from the determined crystal structures. Secondary structure assignments were performed for each PDB id presented in Figure 3.

The α-helix, the most frequently occurring secondary structural element of proteins [22], is a prevalent feature of type III secretion substrates [23,24]. Remarkably, early and middle stage secretion substrates are found to be almost exclusively α-helical (Figure 1). In contrast to β-sheets, α-helices are stabilized through hydrogen bonds formed between adjacent, in the sequence, residues. Hence, the preference for α-helices may reflect the need for local hydrogen bonding when the unfolded polypeptide chain reaches the secretion tunnel, where intact α-helices are probably allowed to be formed (Figure 2). This permits the secretion substrates to become folding competent as soon as they reach the other end of the secretion tunnel. However, when it comes to T3SEs, the use of β-strands is also observed (Figure 3). Despite the occurrence of β-strands, the average preference of T3SEs for α-helices is 10% higher than the average preference in the PDB proteome, while the occurrence of β-strands is approximately 4% lower (Figure 4).

In this review, we will briefly describe the most common α-helical domains that have been observed in T3SEs to date (Table 1). In this context, we will illustrate the variety of α-helical folds associated with T3SS effectors as well as their versatile and diverse roles. We will also discuss the novel functions that these domains sometimes exert as parts of the bacterial protein arsenal and the extent to which they mimic eukaryotic functions.

## 2. The T3SS Gatekeepers Are α-Helical Proteins

All T3SS gatekeepers are α-helical proteins and, among the T3SEs, the first ones to be translocated. They are conserved proteins encoded usually in the T3S gene cluster, charged with an additional important dual role: they mimic T3SEs by occupying a specific position to the export gate and blocking their secretion, while prioritizing the secretion of translocators at the same time [6,25,26]. Depending on the T3S system they serve, gatekeepers consist of one or two polypeptides [26]. They can be classified as later substrates themselves, not only because they are translocated inside the host cell cytoplasm but also because they use a class I T3S chaperone (chaperone of the effectors) for their delivery to the secretion machinery. However, there is a striking difference: this chaperone is not a homodimer but a heterodimer, probably adding to the complexity of this tightly regulated system [6,26]. Gatekeepers have also been found to bind translocator-specific TPR chaperones with their carboxy terminal part, probably facilitating, in this way, the recruitment of the translocators to the secretion gate [27,28].

**Figure 5:**
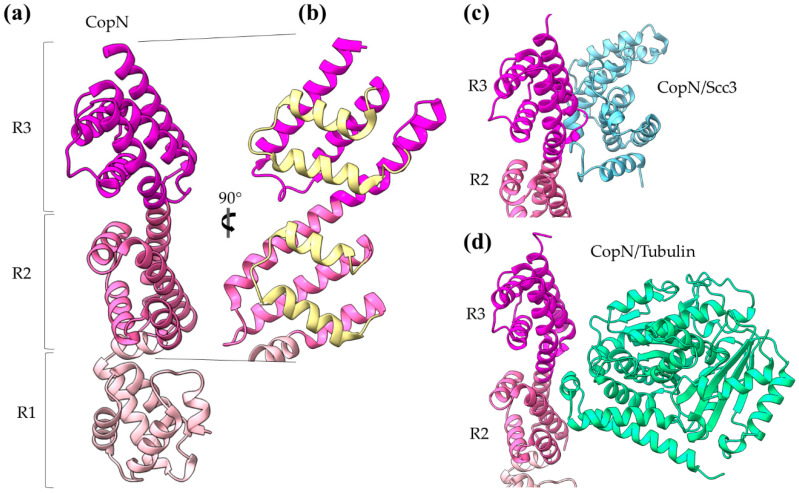
CopN gatekeeper structure. (**a**) The CopN structure consisting of three (R1–R3) structurally homologous 5-helix assemblies, shown in different magenta shades. (**b**) Close-up of the R2 and R3 motifs of CopN. The view of panel (**b**) is rotated 90 degrees in comparison with the view in panel (**a**). The pair of parallel helices is shown in yellow for clarity. (**c**) and (**d**) Part of the CopN structure (only the R3 and R2 motifs are shown) in complex with the Scc3 chaperone of translocators (cyan) and the tubulin (green), respectively.

The *Chlamydia pneumoniae* CopN gatekeeper has been found to inhibit microtubule nucleation when inside the host [29,30]. The whole molecule of CopN is characterized by high plasticity, with both terminal ends completely disordered and absent from the electron density maps of the structures determined by X-ray crystallography. The rest of the protein folds in an elongated tandem repeat of three quite similar five-helix motifs named R1 to R3 (Figure 5) [31]. Despite their structural homology, the motifs share low sequence similarity. Each motif is composed of two sets of parallel helices which are packed together in a crossing angle of approximately 50 degrees. The one set comprises a helix–loop–helix pair (yellow in Figure 5b) and the other a triplex of helices with one (in motifs R1, R3) or two (in motif R2) of them to be shared between successive motifs. The crystal structures of CopN in complex with Scc3 and tubulin display the fundamental role of R2 and R3 motifs in these interactions (Figure 5c,d) [27,29]. The CopN structure resembles the structures of other gatekeepers, such as that of the single chain *Shigella* MxiC and the heterodimeric *Yersinia* YopN/TyeA.

## 3. The Diverse Roles of α-Helical Domains in Animal T3S Effectors

### 3.1. Membrane Docking

**Figure 6:**
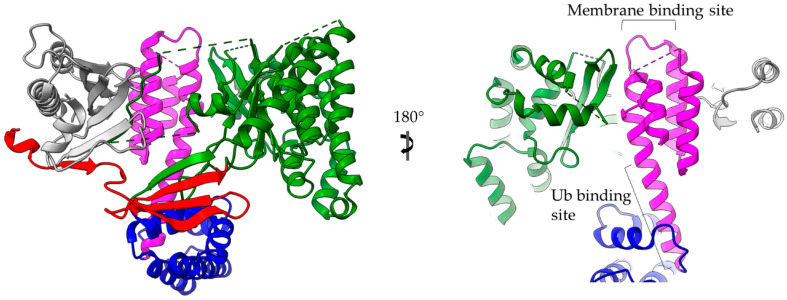
ExoU structure (multi-colored) in complex with the SpcU chaperone (gray). The complex is shown in two views 180 degrees apart. At the right panel the molecule has been clipped for the emphasis to be given on the 4-helix-bundle (magenta). ExoU comprises a highly unstructured N-terminal domain (red), which interacts with the chaperone, a catalytic, patatin-like domain (green), an all-helical bridging domain (blue) and a C-terminal 4-helix domain (magenta). The 4-α-helix-bundle domain is involved in the membrane and ubiquitin binding. The corresponding binding sites are indicated. Ub stands for ubiquitin.

*Pseudomonas aeruginosa* ExoU is one of the most potent T3S bacterial toxins. In agreement with other T3SEs, ExoU’s translocation is ensured via interactions of its partially unfolded N-terminus with a cognate T3S class I chaperone (chaperone of effectors) (Figure 3). The rest of the protein is folded into a patatin-like, α/β, hydrolase domain, an all-helical bridging domain and a C-terminal 4-α-helical bundle (Figure 6) [32,33]. ExoU was the first A2 phospholipase which was identified as a virulence factor translocated via a T3SS [34]. After its translocation inside the host cell, the protein is targeted to the host’s cellular membranes and it causes their irreversible damage. The enzymatic activity is localized on the patatin-like domain and it is activated upon protein interactions with host-originated allosteric factors such as ubiquitin and membrane components, i.e., phospholipids [35,36]. In addition, it has been shown that a Lys residue in the catalytic domain is ubiquitinated, even though this modification has only moderate effect on the protein’s activation.

A dual functional role is attributed to the C-terminal 4-α-helical bundle which is involved in the binding of both the regulatory ubiquitin and the membrane [37,38,39]. Growing evidence suggests that ubiquitin binding and membrane localization act synergistically and induce large conformational changes to this domain [40]. It is well established in the literature that 4-α-helix bundles, i.e., four helices packed together in a way that helix–helix interaction angles are either 20 or 50 degrees, combine remarkable structural plasticity and stability [41,42,43,44,45]. It has also been shown that 4-α-helical bundles are a common structural element used by a variety of bacterial toxins to target the host’s membranes [46]. Two such well-studied non-T3SS transported toxins are the *Pasteurella multicide* toxin (PDB id 2EBF) and the *Clostridium difficile* toxin B (PDB id 2BVL). ExoU has been used as a prototype for investigating the mechanism used by the 4-α-helical bundles to recognize and bind membranes. As many T3SS proteins which are quite flexible in order to ensure some degree of unfolding to facilitate transporting through the narrow needle of T3SSs, ExoU is a highly flexible protein which undergoes multiple conformational changes. It has been thus postulated that the ExoU 4-α-helical bundle undergoes significant conformational changes upon cofactor binding [38,39,41,47]. The different states characterized to date represent different relative orientations of the catalytic domain to the 4-α-helical bundle. According to a recent model, the association of the C-terminus with the membrane is accompanied by a complete unfolding of the 4-α-helical bundle [40,48].

### 3.2. Subverting G Proteins: GAP Activity

**Figure 7:**
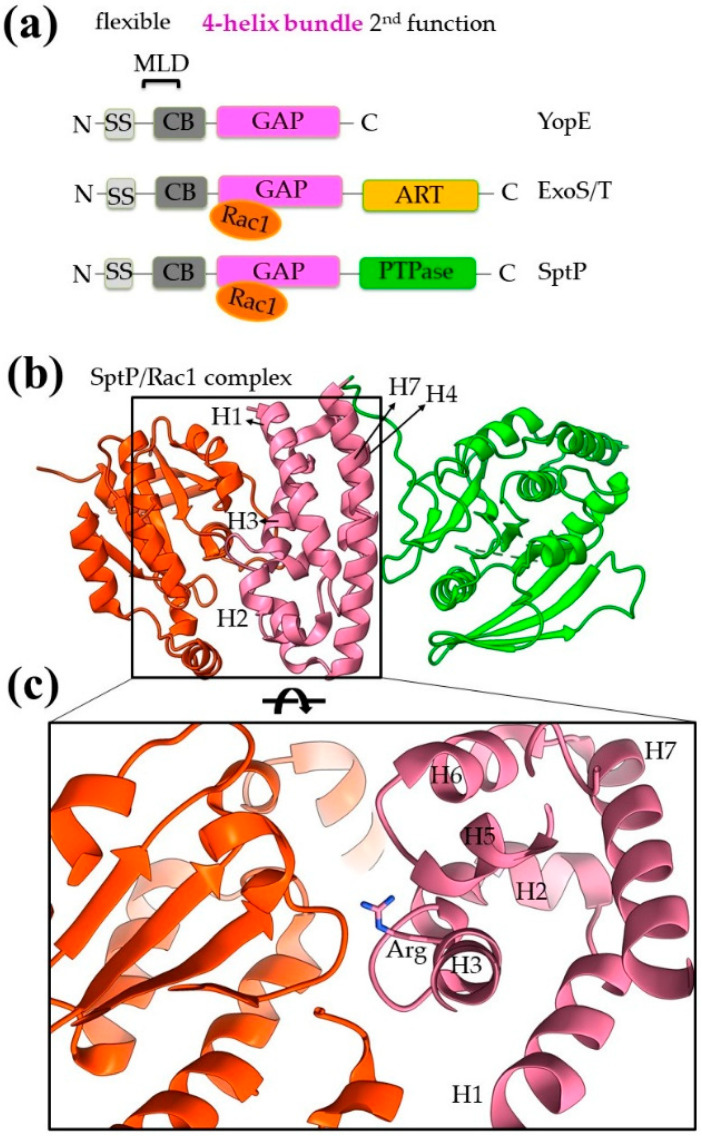
Bacterial structures with GAP activities. (**a**) Schematic comparison of domain architecture of YopE, ExoS/T and SptP. (**b**) The GAP domain is a 4-helix-bundle shown here in the SptP/Rac1 complex structure. Rac1 is shown in orange, the GAP domain in pink and the extra catalytic activity of tyrosine phosphatase (PTPase) in green. (**c**) Close-up of the interaction interface between the Rac1 and GAP domains. MLD stands for membrane localization domain, SS for secretion sequence, CB for chaperone binding.

G proteins are major regulators of cytoskeleton alterations and actin dynamics affecting cell shape, phagocytosis and migration (Rho family), vesicular trafficking (Rab family) and signaling pathways (Ras family) [47,49]. Hence, subverting host G proteins is a highly effective and frequently used pathogenetic mechanism assumed by diverse T3SEs which exert roles of GAPs (GTPase Activating Proteins), GEFs (Guanine nucleotide Exchange Factors) or GDIs (Guanosine nucleotide Dissociation Inhibitors). *Yersinia* YopE, *Pseudomonas aeruginosa* ExoS/T and *Salmonella* SptP have in common a domain with GAP activity (Figure 7a). Whereas the GAP activity of YopE and ExoS/T is believed to be essential for bacterial protection from macrophage attack, SptP GAP activity is involved in the recovery of the infected cells and helps them to regain a normal cytoskeletal architecture after *Salmonella*’s endocytosis.

Except for YopE [50], both SptP and ExoS/T are bifunctional proteins and, apart from the GAP function, they have an additional C-terminal domain with a second catalytic activity (Figure 7a). SptP has an extra tyrosine phosphatase (PTPase) domain, homologous to the corresponding *Yersinia* YopH PTPase domain and other eukaryotic PTPases [51]. ExoS and ExoT are *P. aeruginosa* paralogs with an extra ADP-ribosyltransferase (ART) domain [52,53,54]. All these effectors have a flexible N-terminus, where in addition to the secretion signal and the CBD, a membrane localization domain is also found. This signal leads the T3SEs to the eukaryotic membranes where G proteins are located [55].

In all three effectors, the GAP domain is folded in a very similar 4-α-helical bundle, even though the sequence identity among the domains is less than 30%. The bundle is right-handed, antiparallel with an up-down topology and it is composed by the helices H1, H3, H4 and H7 (Figure 7b). The one side of the bundle (H1 and H3) interacts with the G protein and the other makes stabilization contacts with the following domain, i.e., the SptP PTPase domain (Figure 7b). At one side, the typical 4-α-helical fold is capped with three additional short helices (H2, H5 and H6) which, together with the connecting loops, form an irregular bulge structure. In this case, the bundle provides a stable scaffold from which a variable element, namely the bulge, is folded out and inserts residues into the hydrophobic core of the Rac1 GTPase protein. In this way, the 4-α-helical GAP domain of SptP makes interactions with the GDP and the switch I and II regions of the GTPase. The catalytic arginine, which seems to be universally conserved in the GAP activities, is provided by the middle of H3 to the active site of Rac1 (Figure 7c). A strong electrostatic complementarity has been observed between the interacting sides of the molecules as well as a stabilization of the mobility of the bulge region when it is complexed with molecular partners [51,52]. The 4-α-helical bundle is an unusual GAP domain. The available structures confirm the uniqueness of this fold as compared to eukaryotic GAPs and highlight its similarity with structures of cytochromes [51].

**Figure 8:**
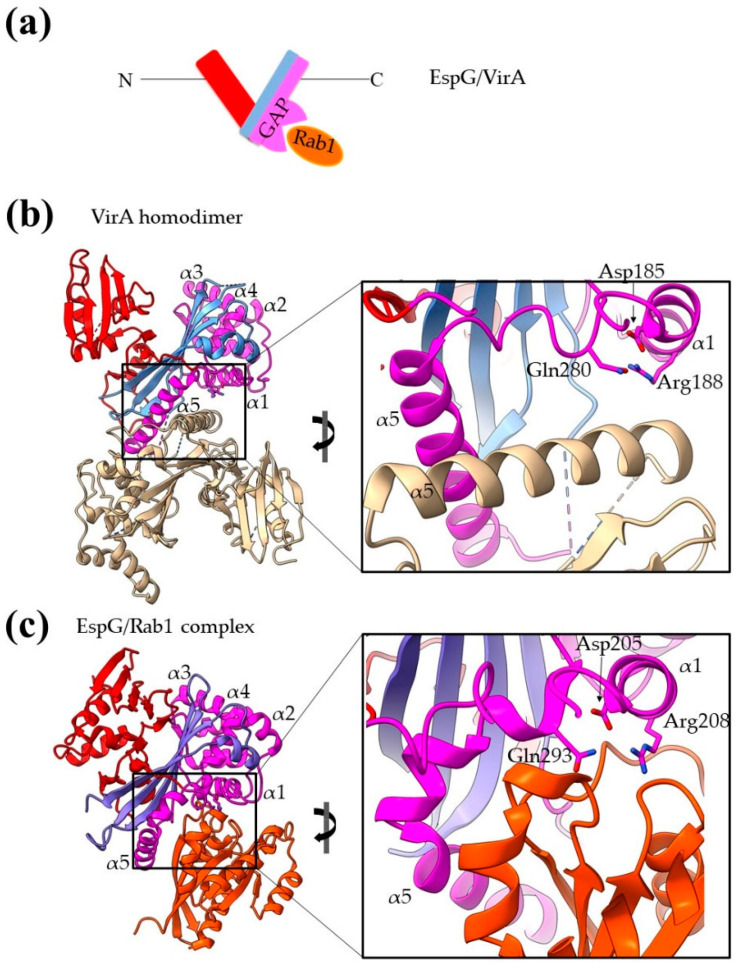
Bacterial structures with GAP activities. (**a**) Schematic representation of the domain architecture of EspG/VirA family members. (**b**) The homodimer of VirA. One monomer is colored white and the other with three different colors highlighting the individual domains. The close-up emphasizes the dimer interface. (**c**) In the EspG/Rab1 complex, the EspG color code is similar to the one used for VirA, and Rab 1 is colored orange. The close-up emphasizes the complex interaction interface and shows that Rab1 binds on a site equivalent to those used for dimerization. MLD stands for membrane localization domain, SS for secretion sequence, CB for chaperone binding.

The EspG family of T3SEs includes the *E. coli* EspG and EspG2, the *Shigella flexneri* VirA and the *Citrobacter rodentium* EspI proteins. Early evidence related the family to the bacterial ability to spread into the dense, host-cell cytoplasm. The identification of an α-tubulin binding site led to the hypothesis that the EspG family controls the cytoskeleton through cleaving tubulin, though no experimental evidence confirmed this hypothesis. On the contrary, structure determination of VirA and EspG [56,57,58] revealed that the proteins share a similar but novel fold without resemblance to proteases (Figure 8). The proteins fold in a V-shaped architecture and each of the two V-arms constitutes an independent protein domain (Figure 8). The N-terminal domain is a flat 4-stranded β-sheet surrounded by short helices and the C-terminal domain is a 6-stranded antiparallel β-sheet whose outer face is shielded by an α-helical domain. The tubulin binding site includes helices α2–α5 (residues 224–315 of VirA). The long, Ser-rich helix α5 is involved in extended dimer stabilization interactions and there is evidence that dimers are the predominant state of the pure protein in solution. Later evidence indicated that VirA/EspG function as GAP proteins of the Rab GTPase [59]. The crystal structures of the complexes show that the interaction interface actually involves the same interface which is involved in the dimer formation, i.e., helices α1 and α5. The non-disposable Arg residue of the GAP activity resides on the helix α1 (Figure 8b,c).

### 3.3. Subverting G Proteins: GEF and GDI Activities

**Figure 9:**
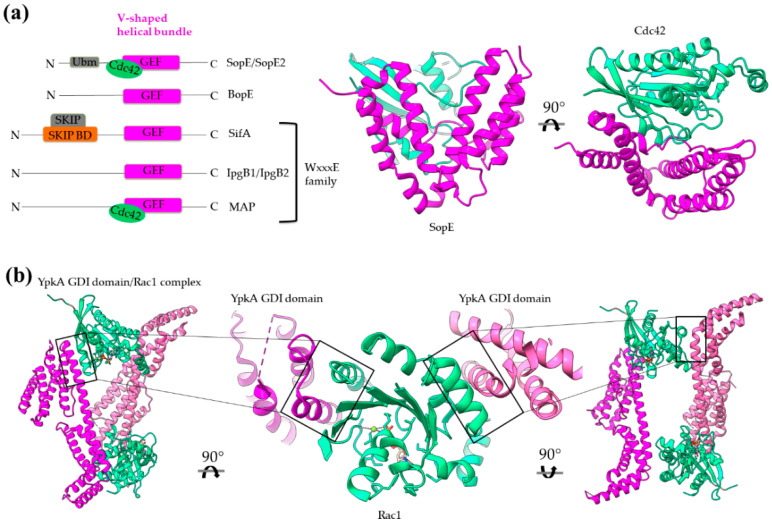
Bacterial structures with GEF (**a**) and GDI (**b**) activities. (**a**) Left: Schematic comparison of the domain architecture of bacterial GEFs. SKIP stands for the host SifA kinesin interacting protein. Right: The GEF domain is a V-shaped helical bundle as it is representatively illustrated by the SopE structure (magenta). The structure of SopE is shown in complex with the host Cdc42 (green, PDB id 1GZS). (**b**)The YopO/YpkA dimer structure (magenta/pink) in complex with Rac1 (green, PDB id 2H7V).

*Salmonella* SPI-1 SopE and SopE2 paralogs (approx. 70% sequence identity), *Burkholderia* BopE as well as *Salmonella* SPI-2 SifA, *Shigella* IpgB1 and IpgB2 and *E. coli* MAP belong to a group of T3S GEFs [60] folded in a unique two-lobe, V-shaped helical domain [61,62,63,64,65]. A 3-helix bundle constitutes each lobe of the structure. The first lobe (starting from the N-terminus) comprises helices 1, 4, 5 and the second helices 2, 3, 6. Hence, there are three extended protein segments which cross the two lobes connecting the helices (Figure 9a). The loop connecting helices 3 and 4 is the catalytic one which contacts the switch I and II of G proteins. The comparison of apo-effectors with G protein complexed effectors shows significant differences in their flexibility, therefore suggesting plasticity and ability for catalytic loop reorientation [62,63,66] as a mechanism of regulating GEF activity. The SifA, IpgB1, IpgB2 and MAP proteins are, in addition, members of the WxxxE family of GEFs. In this subgroup, the specific WxxxE motif, which lies on the beginning of helix 2, is implicated in interactions with the G protein switches (Figure 9a).

*Yersinia* YopO/YpkA is a T3S multidomain effector with dual activity. In the N-terminus of the protein resides a secretion/translocation sequence and a kinase domain, and in the C-terminus resides a domain which mimics, in structure and function, a GDI (Figure 9b). The GDI domain inhibits nucleotide exchange in members of the Rho family (Rac1 and Rho) through a protein complex which is stabilized exclusively through helical interactions (Figure 9b). A functional GDI domain is necessary for *Yersinia* virulence since mutations disrupting the GDI/G protein interface result in impairing cytoskeletal disruption [66].

### 3.4. Acting as Post-Translational Modification Domains

#### 3.4.1. Adenylylation/AMPylation with Fido Domains

Adenylylation or AMPylation is a post-translational modification where proteins are stably modified with AMP contributing significantly to cell signaling [67]. The covalent reversible AMP attachment is achieved through a phosphodiester bond. These all α-helical domains were initially described in *E. coli* as Fic (filamentation induced by cAMP) domains, due to the abnormal growth phenotype observed. They were found later in phages, archaea, eukaryotes as well as many bacterial effectors, not unique to T3SSs [68]. These domains can be further categorized to Fic and Doc (death on curing) domains and are collectively called Fido (Fic and Doc). Remarkably, a third category unique to the T3SE AvrB has been described [68] and is discussed in a following section.

The *Vibrio parahaemolyticus* T3SE VopS covalently modifies a conserved threonine residue on Rho, Rac, and Cdc42 GTPases with AMP. This AMPylation disrupts downstream Rho signaling, leading to the collapse of the actin cytoskeleton and finally cell rounding [69].

The fido core domain contains a central motif conserved in most sequences (HxFx[D/E][A/G]N[G/K]R) and adopts an α-helical fold, arranged as a six-helix up and down bundle. Fido domains usually co-exist with other domains in a single polypeptide chain, creating extensive intramolecular interactions with them. A preference of co-existence with helix-turn-helix (HTH) DNA-binding domains has been observed [68].

#### 3.4.2. Arg-GlcNAcylation

Arginine glycosyltransferases modify mammalian death domain (DD) containing proteins with the addition of *N*-acetylglucosamine (GlcNAc) to arginine residues. Enteropathogenic *E. coli* T3SE NleB blocks the host defense by preferentially modifying the Fas-associated death domain protein (FADD). NleB adopts a typical GT-A glycosyltransferase fold (Figure 3), similar to other known bacterial effectors with a unique helix-pair insertion to hold FADD-DD and the conserved catalytic DxD motif located in the center [70].

*Salmonella enterica* serovar typhimurium can also modify some DD host proteins through arginine-GlcNAcylation to block death receptor-mediated proinflammatory responses. T3SEs SseK3 and SseK1 are specialized to the modification of tumor necrosis factor receptor type 1 (TNFR1) and TNFR1-associated DD (TRADD) protein, respectively [71]. SseK1, SseK3 and NleB structures revealed a very high degree of structural similarity [70].

## 4. The Importance of α-Helical Domains in the (a) Virulence of Bacterial Plant Pathogens

As a defense mechanism against the T3S effectors, many plants have evolved disease resistance (R) proteins. These proteins usually recognize more than one diverse—in sequence and structure—bacterial effectors and activate effector-triggered immunity and/or localized cell death at the site of infection (hypersensitive response, see also Introduction). Thus, plants able to encode R proteins are resistant to the infection. The T3SEs that can be recognized by these plants were historically named avirulent genes (*avr*), mainly due to their ability to elicit the Hypersensitive Response (HR), a typical disease resistance response of plants [2]. Later, it was found that the same effectors are virulent in non-resistant plants. Nowadays, many of them have been renamed to Hrp outer proteins (Hop) in analogy to *Yersinia* outer proteins (Yop). Pathogens are under continuous evolutionary pressure to evade recognition by resistance proteins; therefore, Avr/Hop effectors do not share sequence and structural homology neither with other bacterial effectors nor with known protein classes in the database. Often, they adopt novel structural folds and usually contain a high percentage of α-helices [2,23,72,73,74].

### 4.1. The Helix–Loop–Helix Transcription Activator-Like (TAL) Effectors

**Figure 10:**
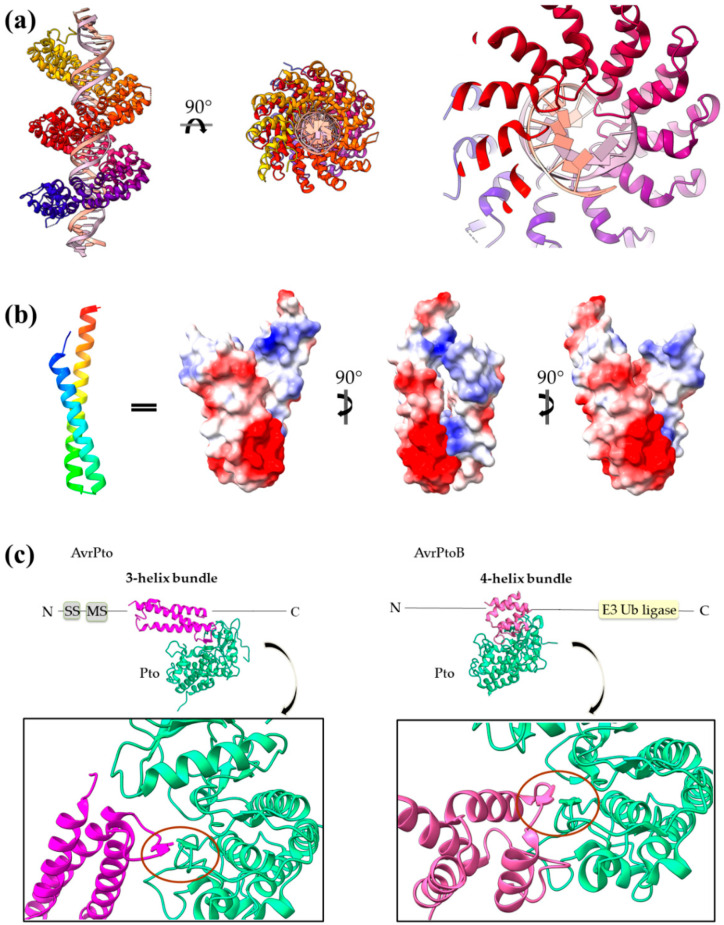
(**a**) PthXo1 protein in complex with DNA (PDB id 3UGM). The structure comprises tandem repeats of a helix–loop–helix motif. The protein is colored from N-terminus (blue) to C-terminus (red). DNA specific recognition and binding occurs through the hypervariable, in sequence, loop of the motif (right). (**b**) The AvrRps4 mature structure is a coiled coil with electrostatically diverse sides (PDB id 4B6X). Red and blue colors on the surface denote negative and positive charge, respectively. (**c**) Each of the AvrPto and AvrPtoB proteins (magenta) interact with the Pto host kinase (green). Left: schematic diagram of the AvrPto protein and close-up of the AvrPto–Pto interaction (PDB id 2QKW). Right: schematic diagram of the AvrPtoB protein and close-up of the AvrPtoB–Pto interaction (PDB id 3HGK).

Transcription activator-like (TAL) effectors are a family of T3SEs which activate transcription in plant cells. Most of the identified to date TAL effectors belong to the *Xanthomonas* genera. These proteins are characterized by a translocation signal, a nuclear localization domain and a central DNA-binding domain. The DNA-binding domain consists of tandem repeats approximately 34 aa long, and each repeat folds in a helix–loop–helix motif (Figure 10a). Although the repeats share high sequence conservation, the loops accommodate two consecutive hypervariable residues which dictate nucleotide specificity for recognition and binding in the target sequence [75,76]. Structure determination of *Xanthomonas* AvrBs3, PthXo1, Hax3 and *Burkholderia rhizoxinica* Bud (BurrH domain) confirms a common architecture able to bind DNA through the loops, in a helical mode following the major groove similarly to the zinc-finger eukaryotic transcription factors [77,78,79].

### 4.2. Coiled-Coils Avirulent Proteins

AvrRps4 is a *Pseudomonas syringae* effector which triggers the Rps4-dependent immunity in *Arabinopsis*. Inside the host cell, AvrRps4 is processed by cleavage, giving a mature molecule folded in an antiparallel α-helical coiled coil [80]. Since there is no evidence for a catalytic site, it is believed that AvrRps4 exerts its avirulent function through interactions with proteinous or non-proteinous host factors. Mutagenesis studies have shown that Rps4 recognition is based on an electronegative AvrRps4 surface [80] (Figure 10b).

### 4.3. Bacterial Avirulence Based on 3- and 4-helix Bundles

*Pseudomonas* AvrPto and AvrPtoB are T3SEs which induce antibacterial immunity in resistant plants, namely, in plants which express the Pto kinase. It has been shown that once in the host, AvrPto interacts with Pto and inhibits its kinase activity by engaging two functionally important Pto loops. Consequently, Pto is rendered unable to interact and repress Prf protein and Prf-mediated defense mechanisms and *Pseudomonas* immunity is induced [81]. Otherwise, in susceptible plants, which do not possess a Pto kinase, the T3SEs target the so called Api (AvrPto-interacting) proteins which are components of signaling pathways. Especially Api2 and Api3 are thought to be small Rho GTPases and Api4 is a myristoyltransferase. AvrPto and AvrPtoB are, in sequence and structure, different proteins and establish quite different interactions with their common Pto target. AvrPto is a single domain, 3-helix bundle [82], while AvrPtoB has two domains, a Pto-interacting 4-helix bundle domain and a U-box E3 ubiquitin ligase domain [83] (see also the following section). In particular, AvrPto assumes a highly flexible conformation with the termini to be the most mobile parts in the structure. Experimental evidence demonstrates that, in solution, the protein dynamically interconverts between a structured and an unstructured state and between monomers and dimers. The low stability and high flexibility of the protein is considered to be a reasonable explanation of the fact that AvrPto does not require a T3S class I chaperone in order to be secreted [82]. The Pto-interacting domain of AvrPtoB comprises four short α-helices and binds Pto via a common and a unique interface in comparison with AvrPto (Figure 10c). However, both effectors use for their interactions elements which fold out of the helical core of the bundles and provide residues to the protein–protein interface, as it was the case for the SptP GAP effector.

### 4.4. Effector Activation Sites Are Formed by α-Helices

When, within the plant cell, the *P. syringae* T3SE AvrB undergoes several modifications in order to become fully functional. Myristoylation, for instance, ensures the correct protein localization to the host plasma membrane. Nucleotide binding and subsequent phosphorylation activate the protein’s function. Activated AvrB targets the immune RIN4 protein and induces its phosphorylation by members of the receptor-like cytoplasmic kinase host family [84]. Modified RIN4 is recognized by the Arabidopsis R protein RPM1 and induces immune response.

Although AvrB was initially thought to be a kinase, its structure determination showed no resemblance to any known kinase [85]. The structure consists of two lobes, a small and a large one. The small lobe is a variable α/β domain required for RPM1 activation. The large is a highly conserved, among the AvrB family members, all-α-helical domain which adopts a six-helix up and down bundle topology, characteristic of Fido domains [68] (see above, paragraph 3.4.1). Despite the lack of a Fic motif in its sequence, AvrB induces the specific phosphorylation of a Thr residue in RIN4 [86]. Fido domains have been found to covalently modify amino acids containing a hydroxyl group, by the addition of phosphate-containing chemical groups, such as: AMP, UMP, phosphocholine and phosphate [87]. Consistent with this function, the domain accommodates a nucleotide-binding pocket which is formed by several conserved residues [88]. It was shown that mutations of nucleotide-binding site residues completely abolish AvrB-mediated RPM1 response.

### 4.5. The Multiple Facets of Enzymatic Regulation by α-Helices

**Figure 11:**
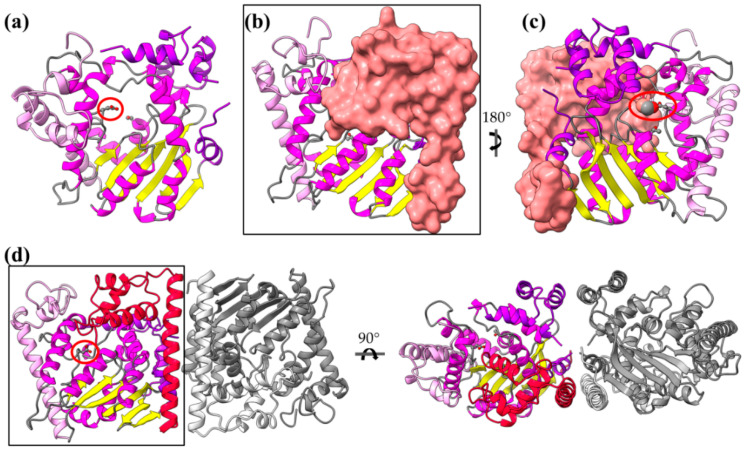
The structure of AvrRxo1-ORF1 and its complex with the AvrRxo1-ORF2 chaperone (PDB id 4Z8V). (**a**) The AvrRxo1-ORF1 monomer consists of a major, middle α/β domain (helices are colored in magenta) and two flanking domains, whose helices are colored pink and violet, respectively. (**b**,**c**) The 1:1 complex of AvrRxo1-ORF1/AvrRxo1-ORF2 in two views, 180 degrees apart. AvrRxo1-ORF2 is shown in surface representation. (**d**) The 2:2 complex of AvrRxo1-ORF1/AvrRxo1-ORF2 in two views, 90 degrees apart. One dimer is gray and the other is colorful, consistent with colors in (**a**). AvrRxo1-ORF2 is shown in red cartoon.

AvrRxo1-ORF1 is a *Xanthomonas* effector recognized by the Rxo1 R protein of resistant rice plants [89]. The protein comprises a major, middle domain similar to a T4 polynucleotide kinase (T4pnk) (Figure 11a). T4 polynucleotide kinases phosphorylate the 5′-OH terminus of nucleic acids using ATP or other nucleoside triphosphates. The protein seems to form stable dimers based mainly on helical interactions provided by the flanking N- and C-terminal ends. It has been shown that AvrRxo1-ORF1 suppresses bacterial growth and that this toxicity depends on the T4pnk domain and the predicted catalytic residues residing in this domain, i.e., the substrate-binding Asp193 and the ATP-binding Thr167 (Figure 11a, residue in ball-and-sticks representation). Moreover, AvrRxo1-ORF1’s bacteriostatic activity is suppressed by the AvrRxo1-ORF2 protein, which forms a complex with AvrRxo1-ORF1 (Figure 11b,c) in a stoichiometry of 2:2 (Figure 11d). It is believed that AvrRxo1-ORF2 is a chaperone which inactivates AvrRxo1-ORF1 when it is within the bacterium. AvrRxo1-ORF2 is a helical, two domain protein. One long helix interacts with and is stabilized on the AvrRxo1-ORF1 dimer (Figure 11d). The rest of the helices form a globular domain used to block the substrate binding site (Figure 11b,c). As it is shown in Figure 11c, a direct interaction is formed between an AvrRxo1-ORF2 Ser residue and the predicted substrate-binding AvrRxo1-ORF1 Asp residue.

**Figure 12:**
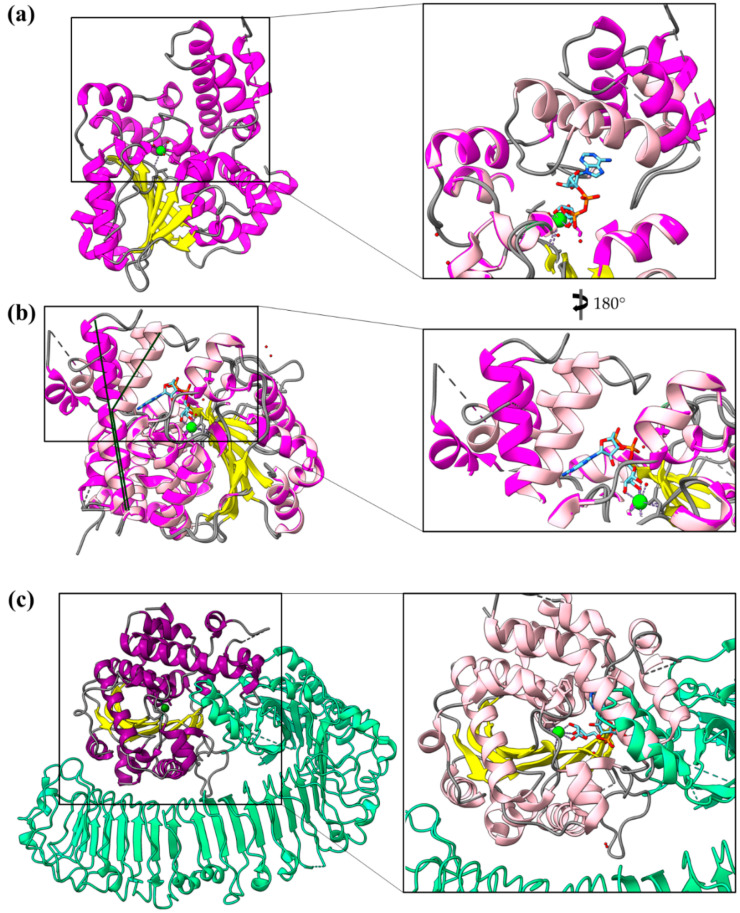
XopQ structure and conformational changes. (**a**) The structure of *Xanthomonas* XopQ is a Rossmann fold implemented with a mobile helical segment able to function as a lid of the active site upon substrate binding (PDB id 4KL0). (**b**) Closure of the active site is achieved via a bend which occurs in the middle of a long helix (PDB id 4P5F). The dark and light tones of magenta indicate the open and closed conformations of XopQ protein, respectively. (**c**) Left panel: The structure of XopQ (magenta/yellow) in complex with the Roq1 (green) indicates that the latter inserts a helical segment into the active site cleft of XopQ (PDB id 7JLU). Right panel: Close-up of the complex. In the structure, the XopQ has been substituted by a superimposed closed conformation. It is evident that the Roq1 helix insertion overlaps with the XopQ helical-lid in the closed conformation.

*Xanthomonas campestris* XopQ is an effector with homologs in *Xanthomonas oryzae*—XOO4466, 94% sequence identity—and *Pseudomonas syringae*—HopQ1-1, 61% sequence identity. Crystal structures show that XopQ family members are predominantly alpha helical proteins with a central Rossmann fold which accommodates the catalytic center and the Ca^2+^ binding site (Figure 12a), resembling the total fold of the nucleoside hydrolases [90,91]. Nevertheless, a protruding helical segment in the vicinity of the active site, which is involved in ligand-induced conformational changes and determines two distinct states (open and closed) of the active site, is unique in the XopQ family (Figure 12a,b) and indicates the functional distinction of the families. Crystal structure determination identified an adenosine diphosphate ribose molecule bound in the XopQ active site and correlated the binding of the substrate with a movement of the helical segment towards the active site, therefore functioning as a helical lid [91].

XopQ is recognized by the plant Roq1 R protein, which is a nucleotide binding leucine-rich repeat factor with a Toll-like interleukin-1 receptor domain [92]. The XopQ/Roq1 complex (Figure 12c) shows that Roq1 recognizes XopQ by inserting a helical segment into the active site. Thus, Roq1 binds and blocks XopQ activity [93]. The right panel of Figure 12c displays that the helical insertion of Roq1 superimposes with the XopQ helical lid in the closed conformation.

## 5. Marginal Resemblance of T3SE Folds to Functionally Related Proteins

Although many T3SEs adopt entirely novel folds, there are few examples of effectors which retain a degree of similarity with previously characterized protein classes so that functional and structural comparisons can be performed. Here, we discuss the NleC/GtgA metalloprotease, the NleH kinase and the atypical LRR-NEL families of T3SEs in relation to their protein analogs and the evolution of α-helices.

### 5.1. The NleC/GtgA Family of T3S Zinc Metalloproteases

Members of the NleC/GtgA family of T3SEs are zinc-dependent endopeptidases which degrade subunits of the NF-κB complex, and therefore inhibit host innate immune responses. The family contains at least six members, namely *EPEC* NleC, *EHEC* NleD, *Salmonella enterica* GtgA, GogA, PipA and *Ralstonia Solanacearum* RipAX2. The determined structures of NleC and GtgA [94,95,96] show that the family adopts a new fold which only distantly resembles the family of zinc metalloproteases. Indeed, the NleC/GtgA T3SEs contain a conserved zinc metalloprotease catalytic motif HExxH, which similarly to zincin zinc proteases is accommodated to an active site helix. Two more helices around the active site helix provide catalytic residues and build up the active site cleft configuration, which also displays common features with the zincin zinc proteases. Therefore, the NleC/GtgA family of T3SEs have been classified into a distinct class within the zincin fold superfamily of zinc metalloproteases [94,95].

A Dali search of the PDB with the structure of NleC (PDB id 4Q3J) revealed the *E. coli* BepA metalloprotease (PDB id 6SAR, [97]) as its closest, no-T3SE relative (Z-score = 5.3 and rmsd = 3.5 Å). A noticeable difference between the proteins is the distortion of a Ψ-loop β-sheet motif in the T3SE [98]. The Ψ-loop is a mixed beta-sheet of three β-strands arranged such that the central strand is parallel to the N-terminal strand and is located in the proximity of the active site cleft. It was proposed that this distortion of the motif may reflect evolutionary differences dictated by the necessity for NleC to cross the T3SS channel.

The observation that the T3SE metalloproteases cleave the substrate in loops which are essential for DNA binding formulated the hypothesis that they use a DNA mimicry mechanism to specifically recognize, bind and cleave their substrates. Detailed structural and mutagenesis analysis confirmed that the NleC and GtgA proteins mimic the structure and electrostatics of DNA. Furthermore, it was shown that the NleC proteolytic activity is inhibited in the presence of DNA, consistent with the hypothesis that both molecules occupy the same substrate protein site [94,95,96].

### 5.2. The NleH Family of T3S Kinases

NleH is a new family of T3SE kinases which retain a minimal kinase fold. The NleH family includes three well-studied members, namely *EPEC* NleH1, *EHEC* NleH2 and S*higella* OspG. The host NF-κB pathway has been identified as the primary target of the family, which leads to blocking apoptosis. Moreover, it was observed that *in vitro* the proteins exhibit a low kinase activity which is enhanced upon interaction with the host ubiquitination network. The structure determination of NleH and OspG revealed that the proteins comprise a N-terminal domain which, in part, is intrinsically unfolded and a C-terminal, mainly helical kinase-like domain. The T3SE kinase domain misses several segments of the classical kinase fold, including the terminal helices and the activation loop [99,100,101]. The OspG structure in complex with the ubiquitin (Ub) conjugated E2 ligase reveals the sites of interaction and implies the way that the Ub~E2 conjugate activates the T3SE kinase activity by stabilizing the proposed P-loop of the enzymes [101].

### 5.3. The LRR-Containing T3S Effectors

The Leucine-Rich Repeat (LRR) fold is composed by the tandem repeat of the β-α-β motif, forming a horseshoe-like fold with a concave and a convex surface. The β-strands are systematically arranged in the concave site, and the α-helices, which in some cases are 3_10_ helices, i.e., they have only three residues per turn, are located at the convex site. Although the fold is mainly found in eukaryotes, there are also bacterial LRR proteins. The LRR-containing T3SEs constitute a distinct subfamily because they have the shortest repeating unit, with 20 to 22 amino acids, among all known LRR superfamily members. The consequence of the short repeating unit is that the strand which comprises the convex site has no helical context, but instead adopts an extended conformation. The T3SE LRR is a substrate binding domain [102,103,104].

To date, the LRR-containing T3SE family comprises two protein groups. The one is represented by the nuclear YopM protein [105,106], and the other by the IpaH/SspH (LRR-NEL) proteins which are novel bacterial E3 ligases. The LRR-NEL proteins are composed of an N-terminal putative substrate-binding LRR domain and a C-terminal catalytic all-α helical NEL (Novel E3 Ligase) domain. NEL is a novel bacterial E3 ligase which is distinct from the known eukaryotic HECT or RING domains (for a structural comparison, please see [107,108]). The NEL domain uses a catalytic Cys residue located in a loop and its activity is regulated by conformational flexibility and domain rearrangements [21,104,109]. In *Shigella* IpaH9.8, the catalytic Cys can be involved in the formation of a disulfide bond, which makes the loop helical and converts the two adjacent helices to one long helix [110].

## 6. Discussion

T3SE families adopt novel folds to target eukaryotic functions. These folds comprise a high helical content, which possibly reflects the specific requirements from T3SS effectors. In particular, effectors must (i) be able to be easily unfolded, (ii) cross the narrow T3S channel, (iii) be highly folded as soon as they will be found inside the host cell, in order to evade the host defense mechanisms, and (iv) display functional competence and structural plasticity in their final destination. α-helices can optimally fulfil these requirements. The main advantage of α-helices when compared to β-sheets is the local character of required hydrogen bonding, which stabilizes this secondary structural element. T3SEs are probably allowed to form α-helices in the secretion tunnel after passing the secretion gating mechanism [21]. These pre-folded parts may render the T3SEs highly folding competent by acting as nuclei that promote the rapid overall folding of the polypeptide chain once inside the host cell cytoplasm. α-helical domains may achieve high folding/unfolding rates, as it has been demonstrated in the literature [109,111,112,113,114].

There are multiple ways that the T3SE helical domains support the pathogenic requirements for specific catalytic activity, tight regulation and structural plasticity: (i) Helical assemblies form structural scaffolds from which other elements, for instance, bulges and loops, are protruding and achieve either protein–protein interactions or provide catalytically important residues. (ii) Individual α-helices can be actively involved in the function by, for example, providing catalytic or metal binding residues. (iii) α-helices assemble together to form binding sites of the required physicochemical properties concerning, for example, hydrophobicity or electrostatics.

The 4-α-helix bundles appeared several times in the T3SEs we discussed earlier, serving a variety of functions. We can distinguish two main interaction patterns that this motif uses for function: it either provides an extra element which folds out of the main helical assembly or one of the four main helices is directly involved in the function. The AvrPtoB and the YopE family of T3SE GAPs use bulges and loops to make interactions with their protein partners and even to provide significant residues to interaction sites, whereas the ExoU family uses its 4-α-helix bundle in a dual way. At least one of the loops of the bundle anchors the protein to the membrane, while one of the bundle helices makes partial interactions with a regulatory ubiquitin molecule and contributes to the protein’s activation.

The same concept also applies for other helical assemblies. Indeed, 3- and 2-helix bundles, namely helix–loop–helix motifs and coiled coils, are frequently used by T3SEs. AvrPto uses bulges protruding from its 3-helix bundle to make interactions with the Pto, and the modular TAL effectors use the loops of a repeated helix–loop–helix motif to achieve specific DNA binding. The SopE family of GEFs folds in a novel V-shaped assembly composed of two 3-helix bundles and provides the catalytic residue from one of the three connecting loops. Similarly, the catalytic Cys of the NEL T3SE E3 ligases, which are composed of 12 to 14 helices, resides on a loop connecting two helices.

NEL domains can also provide an example of regulation by α-helices. For at least a member of the family, it has been shown that the loop where the catalytic residue resides can be re-folded in a helix dependent on the redox conditions. More trivial examples of regulation based on helices include T3SEs which possess flexible helical caps able to oscillate around an active/binding site. Helices can also constitute barriers which block and isolate functional sites.

In a different mode of action, the coiled coil of AvrRps4 seems to use the electrostatic properties of its surface to make interactions with protein factors. Likewise, the T3SE zinc metalloproteases mimic DNA electrostatics to create a suitable active site cleft in order to specifically bind their substrate. In addition, these enzymes use an active site helix which provides several residues important for the catalysis. The same is also true for the VirA family of T3SE GAPs we described. The universally used for GAP action Arg residue is provided by an α-helix, which also participates in the substrate binding.

The gating mechanism for unfolded protein translocation has been revealed recently, as a substrate-engaged needle complex structure, which it has been solved and analyzed in high enough resolution [21]. The far longer secretion tunnel, however, is shaped to a right-handed helix with a minimal inner diameter around 13 Å, a space large enough to accommodate α-helices (Figure 2c,d). The substrate density itself, as seen inside the lumen, is comparable to low resolution tubular densities of α-helices, further supporting this view. Although a fully unfolded polypeptide is probably needed to bypass the unidirectional portals found on the secretion core and entrance gate of the T3S machinery, it is possible that α-helices might refold when the polypeptide reaches the needle tunnel (Figure 2). The preference towards α-helices might ensure the fast refolding of the substrates when on the other side of the tunnel. This could also be advantageous for a fast-growing needle.

Due to the highest sequence conservation of the needle protomers on their C-terminal part, the part that is directed to the interior of the needle, we can safely hypothesize the existence of a common translocation mechanism adopted by several T3SS families. The *E. coli* T3S filament is another unique structure, arising from the evolutionary need of the bacterium to penetrate the intestinal mucus layer, which we can gain valuable insights from. This filament also seems to possess a large enough lumen (22 Å) probably capable of forming a seamless conduit with the T3S needle tunnel as both the architecture of the filament lumen and its electrostatic properties resemble the ones of the T3SS needle tunnel [115]. Unfortunately, we lack high resolution data from the much longer pilus (several μm) of the plant pathogenic T3SSs that penetrates the thick plant cell wall. However, it is quite possible that plant associated T3SSs are also following the same, still not fully understood, T3S translocation mechanism.

There is a clear preference towards α-helices in type III secretion substrates. Remarkably, the early and middle substrates are exclusively all α-helical, while T3SEs possess a higher than usual percentage of α-helical content. This tendency probably reflects an evolutionary pressure related to the common fate of all the substrates: travel through the narrow secretion channel.

## 7. Materials and Methods

Representative T3SEs were selected through the BastionHub catalogue [116] for type III secreted effectors with Protein Data Bank (PDB) records and unique folds. Data mining from the above databases took place on January 2021.

Secondary structure assignments on known domains, as shown in Figure 4, were performed using the hydrogen bond estimation algorithm DSSP (Define Secondary Structure of Proteins) through the 2 Struct server [117].

All figures presented in this work were originally prepared using experimental data deposited with Protein Data Bank (PDB) or Electron Microscopy Data Bank (EMDB) and the molecular visualization program ChimeraX [118,119,120]. Likewise, all surface renderings were performed in ChimeraX. Superposition of atomic structures was performed using the matchmaker function of ChimeraX. Molecular maps of a given resolution, as indicated in figure legends, were prepared using the molmap function of ChimeraX. Calculation of the cavity representing the T3SS needle tunnel, as displayed in Figure 2, was performed in 3V [121] using a high grid resolution. To define the excluded surface, two probes of 10 and 3 Å were used. The final surface produced was smoothed in ChimeraX.

## Figures and Tables

**Table 1:** List of the α-helical T3S effectors discussed in this paper.

General Function	T3SEs	Specific Host Target
Gatekeepers	*Chlamydia pneumoniae* CopN *Shigella* MxiC, *Yersinia* YopN/TyeA (heterodimer)	Scc3, tubulin
Membrane docking	*Pseudomonas aeruginosa* ExoU	membrane
GAP activity	*Salmonella* SptP, *P. aeruginosa* ExoS/T *E. coli* EspG, EspG2, *Shigella flexneri* VirA, *Citrobacter rodentium* EspI	Rac1 Rab
GEF activity	*Salmonella* SPI-1 SopE, SopE2, *E. coli* MAP *Burkholderia* BopE, *Salmonella* SPI-2 SifA, *Shigella* IpgB1, IpgB2	Cdc42
GDI activity	*Yersinia* YopO/YpkA	Rac1
PTM activity	*Vibrio parahaemolyticus* VopS (AMPylation) *P. syringae* AvrB *E. coli* NleB, *Salmonella* Ssek3, Ssek1 (Arg-GlcNAcylation)	Rho, Rac, Cdc42 RIN4 DD proteins
Transcription activation	*Xanthomonas* AvrBs3, PthXo1, Hax3, *Burkholderia rhizoxinica* Bud	DNA
Host immunity activation in resistant plants	*Pseudomonas syringae* AvrRps4 (suppress immunity in susceptible plants, chloroplast localization is required) *Pseudomonas* AvrPto, AvrPtoB *Xanthomonas* AvrRxo1-ORF1 (T4-polynucleotide kinase) *Xanthomonas campestris* XopQ (suppress immunity in susceptible plants)	Pto * (Api) Rxo1 * Roq1 * (14–3-3)
Zinc metalloproteases	*EPEC* NleC, *EHEC* NleD, *Salmonella enterica* GtgA, GogA, PipA *Ralstonia solanacearum* RipAX2	NF-κB
Kinases	*EPEC* NleH1, *EHEC* NleH2, *Shigella* OspG	NF-κB
Novel E3 Ligases	*Shigella* IpaH, *Salmonella* SspH	Ub network

* Plant R (resistance) proteins in resistant plants. Abbreviations used in this table and throughout the paper: Api, AvrPto interacting; ART, ADP-Ribosyltransferase; Avr, Avirulence; CBD, Chaperone Binding Domain; DD, Death Domain; EHEC, Enterohemorrhagic Escherichia coli; EPEC, Enteropathogenic *E. coli*; GAP, GTPase Activating Protein; GEF, Guanine nucleotide Exchange Factor; GDI, Guanosine nucleotide Dissociation Inhibitor; GlcNAc, N-acetylglucosamine; HR, Hypersensitive Response; LRR, Leucine-Rich Repeat; MLD, Membrane Localization Domain; NEL, Novel E3 Ligase; NF-κB, Nuclear Factor Kappa B; ORF, Open Reading Frame; PDB, Protein Data Bank; PMF, Proton Motive Force; PTM, Post-Translational Modification; PTPase, Protein Tyrosinephosphatase; R protein, Resistance protein; SPI, Salmonella Pathogenicity Island; SS, Secretion Sequence; TALE, Transcription Activator-Like Effector; TPR, Tetratrico-Peptide Repeats; T3SS, Type 3 Secretion System; Ub, Ubiquitin.

## Data Availability

Not applicable.
